# Using eHealth Technologies: Interests, Preferences, and Concerns of Older Adults

**DOI:** 10.2196/ijmr.4447

**Published:** 2017-03-23

**Authors:** Patrick Ware, Susan J Bartlett, Guy Paré, Iphigenia Symeonidis, Cara Tannenbaum, Gillian Bartlett, Lise Poissant, Sara Ahmed

**Affiliations:** ^1^ School of Physical and Occupational Therapy McGill University Montreal, QC Canada; ^2^ Institute of Health Policy, Management and Evaluation University of Toronto Toronto, ON Canada; ^3^ Division of Clinical Epidemiology McGill University, and McGill University Health Centre Montreal, QC Canada; ^4^ Research Chair in Digital Health HEC Montréal Montreal, QC Canada; ^5^ Centre de Recherche, Institut Universitaire de Gériatrie de Montréal Université de Montréal Montreal, QC Canada; ^6^ Department of Family Medicine McGill University Montreal, QC Canada; ^7^ School of Rehabilitation Université de Montréal Montreal, QC Canada; ^8^ Centre de recherche interdisciplinaire en réadaptation (CRIR) Institut de réadaptation Gingras-Lindsay-de-Montréal Montreal, QC Canada; ^9^ Centre de recherche interdisciplinaire en réadaptation (CRIR) Constance-Lethbridge Rehabilitation Center Montreal, QC Canada

**Keywords:** Internet, telemedicine, self care, chronic disease

## Abstract

**Background:**

The Internet and eHealth technologies represent new opportunities for managing health. Age, sex, socioeconomic status, and current technology use are some of the known factors that influence individuals’ uptake of eHealth; however, relatively little is known about facilitators and barriers to eHealth uptake specific to older adults, particularly as they relate to their experiences in accessing health care.

**Objective:**

The aim of our study was to explore the interests, preferences, and concerns of older adults in using the Internet and eHealth technologies for managing their health in relation to their experiences with the current health care system.

**Methods:**

Two focus groups (n=15) were conducted with adults aged 50+ years. Pragmatic thematic analysis using an inductive approach was conducted to identify the interests, preferences, and concerns of using the Internet and eHealth technologies.

**Results:**

Five themes emerged that include (1) Difficulty in identifying credible and relevant sources of information on the Web; (2) Ownership, access, and responsibility for medical information; (3) Peer communication and support; (4) Opportunities to enhance health care interactions; and (5) Privacy concerns. These findings support the potential value older adults perceive in eHealth technologies, particularly in their ability to provide access to personal health information and facilitate communication between providers and peers living with similar conditions. However, in order to foster acceptance, these technologies will need to provide personal and general health information that is secure, readily accessible, and easily understood.

**Conclusions:**

Older adults have diverse needs and preferences that, in part, are driven by their experiences and frustrations with the health care system. Results can help inform the design and implementation of technologies to address gaps in care and access to health information for older adults with chronic conditions who may benefit the most from this approach.

## Introduction

Canadians aged 65 years and older currently represent 14% of the population, and this proportion is expected to increase to approximately 25% by 2036 [1]. In 2011, 3 of 4 Canadian seniors reported having at least one chronic condition, and 1 in 4 reported having 3 or more chronic conditions [2]. The burden of chronic disease on the health care sector and society as a whole has an effect on increased costs, reduced patient function, and poorer quality of life [3]. There is also a trend toward a higher incidence of chronic diseases such as diabetes in younger age groups. For example, those in the 45 to 64 years age range represented almost half (48%) of incident cases of diabetes in Canada in 2009 [4].

Ongoing management of chronic conditions requires considerable effort, time, and energy by patients and often family members [5]. This is largely because what individuals do between clinic visits will impact their health far more than what happens in the doctor’s office [6]. eHealth technologies such as personal health records (PHRs) and remote monitoring tools can potentially support self-management efforts on a wide scale. For example, PHRs linked to electronic health records (EHRs) give individuals secure access to their personal health information (PHI), and in some cases provide direct access to their care team. For people living with chronic diseases, up-to-date health information and easier access to providers can empower them to learn more about their health conditions, take more responsibility to better manage their health, communicate more efficiently and effectively between visits, and ultimately experience better health outcomes [6].

Recent estimates have shown that 22% (nearly 1.5 billion) of people worldwide use the Internet regularly [7], with older adults representing the group with the highest rates of increase in the past decade [8]. However, increased Internet use has not yet translated into greater use of eHealth technologies in chronic disease populations [9,10]. One challenge has been to design systems that are accepted and used effectively by older adults, which should include features for ongoing monitoring, interpretation of PHI, and recommendations [9,11-14]. Sociodemographic factors including age, sex, and socioeconomic status [15-24], and a lack of user-friendly interfaces have been identified as key barriers to eHealth uptake in older populations [25]. However, no studies have qualitatively explored the relationship between older individuals’ experiences with the health care system and their needs and preferences for using the Internet and eHealth technologies for managing their health. These experiences are important to consider as this age group represents the highest users of the health care system [1] and have the most to gain from tools that can facilitate the management complex comorbidities often found in aging populations. Preferences for entering, maintaining, and disclosing portions of their medical record, and considerations required to adapt Internet resources and eHealth technologies to sustain interest over time remain understudied [26]. Although identified barriers include limited computer literacy, computer anxiety, cognitive impairment, health literacy, and physical impairments [27], features that help to both motivate and sustain self-management efforts in older adults remain largely unexplored.

Therefore, the goal of this study was to learn more about the interests, preferences, and concerns of adults aged 50+ years regarding use of the Internet and eHealth technologies to manage their health in relation to their experiences with the current health care system. The age range was chosen to focus on a segment of the population who have or are at higher risk of developing chronic conditions.

## Methods

### Definitions

We defined eHealth as any technology which enables the performance of a health-related task, either accessible on the Web or enabling a Web-based information exchange (eg, health portals, software connecting to the Internet, and mobile apps). Health-related tasks were broadly defined as any activity related to health behavior change, enabling health information exchange, or health-related administrative-type tasks (eg, e-booking of medical appointments).

### Interview Guide Development

First, a scoping review was conducted to identify knowledge gaps around factors that impact use of the Internet and eHealth technologies. The following databases were searched: Cochrane (1977-2012), MEDLINE (1970-2012), EMBASE (1980-2012), and CINAHL (1970-2012) using combinations of MeSH terms and keywords including chronic disease, technology, self-efficacy, health attitudes, and health promotion. The literature search yielded a list of candidate domains that were reviewed by content experts (IS, SA) for relevance. Focus group questions were generated using Kruegar guidelines [28] and included the following: (1) Have you ever accessed your health record/medical chart? (2) Do you know whether you have access to your personal health record? (3) Do you think you would use a website where you could login and access your electronic health record/medical chart? (4) How would you feel about sharing your health information and your health problems with your clinical team via this web portal? (5) How would you feel about receiving advice based on your symptoms via a web portal? and (6) What would further entice you to manage your health through the use of an electronic health chart?

### Recruitment

To recruit participants, posters were placed in rehabilitation clinics and community organizations in a large urban city in Quebec, Canada inviting adults aged 50 years and older to participate. Participants both with and without chronic diseases were included to explore the use of the Internet and eHealth technologies for the prevention and management of chronic diseases.

### Focus Groups

Two focus groups were conducted, each lasting 2 hours, which were led by a trained member of the research team. An assistant was present to take notes, provide clarifications, and summarize key points throughout the session. All sessions were audio taped.

### Data Analysis and Theme Development

Audio files were transcribed and compared with the original recordings to verify accuracy. Three reviewers (SA, SB, and PW) conducted a pragmatic thematic analysis [29] independently to identify themes [29]. Themes were compared and differences were discussed and reconciled. Similar subthemes were combined to provide an encompassing theme.

## Results

### Study Participants

Participants were 15 adults who were mostly female (73%, 11/15) with a mean (SD) age of 67 (10); see Table 1). Eight (53%, 8/15) had completed high school and the remaining were university educated. Almost all (87%, 13/15) reported regularly using the Internet at home or elsewhere (eg, public library) and 12 (80%, 12/15) reported having 1 or more chronic diseases.

### Focus Group Themes

Five themes were identified: (1) Difficulty identifying credible and meaningful sources of information on the Web; (2) Ownership, access, and responsibility for medical information; (3) Peer communication and support; (4) Opportunities to enhance health care interactions; and (5) Privacy concerns. Themes are discussed in more detail in the following section.

#### Difficulty Identifying Credible and Relevant Information on the Web

All participants expressed frustration with finding credible and relevant information on the Web regarding their health conditions. Most felt overwhelmed by the volume of information available and had difficulty identifying whether information was credible or not.

You go to Google and you have about twenty different things. Which one is the best one to go to?

P14

One thing I’ve found is that there is so much absolute garbage out there. And that’s what I find difficult in dealing with my health situation...What is an online medical dictionary that’s correct? If you’re sick, no one’s going to sit with you and tell you this is where you find (the information).

P4

Even when users were confident that the information was trustworthy, they noted that it was often not presented in a meaningful way or in ways that made it easy to understand. They felt that information needed to be presented in a user-friendly way and placed into context so that individuals can understand what it means and how to act on it.

I like things boiled down. I want the essentials. If I type in a medication and ask for the side effects, I don’t want it (the Internet site) to give me the runaround.

P11

If we take the example of high blood pressure, sometimes they will say, “you are 135 over 80,” people don’t know what that means. Is this something that needs to be checked?

P8

**Table 1:** Characteristics of focus group participants.

Participant	Sex	Age group (years)
P1^a^	Male	50-59
P2	Female	50-59
P3	Female	60-69
P4	Male	50-59
P5	Female	60-69
P6	Female	50-59
P7	Female	>80
P8	Female	>80
P9	Female	70-79
P10	Male	60-69
P11	Female	>80
P12	Female	>80
P13	Female	>80
P14	Male	60-69
P15	Female	60-69

^a^P: participant.

#### Ownership, Access, and Responsibility for Medical Information

All participants expressed a desire to have greater access to their medical records and many viewed themselves as the ultimate owners of their medical information. However, some expressed frustration at being treated as if they did not have a right to access their information when needed. Most reported encountering frequent barriers to gaining ready access to PHI through usual channels (requesting results of medical tests or copies of medical records, and so on.)

Opinions varied as to who should be responsible for managing their health information. Two people expressed interest in assuming responsibility for compiling their medical information so that it could be shared with all care providers, including the ability to edit the data when needed. This desire to act as gate-keeper was presented as an important strategy to increase both the continuity and quality of the health care they received because new providers often did not appear to have access to their existing medical record.

When I go to my family doctor, who’s supposed to combine everything in a way that there is everything in my charts from over the past 20 years? So I highlight and I make specific copies after consult(ing) with other specialists.

P3

There was a time when I went to the Emergency (Department). I had my records with me, so I gave them the copy, and they lost it. Within a day only (they were lost)...but luckily I had kept my copy. They...put it in the file, but they said, “Keep your copies with you in case you need it again.” I have my whole medical record.

P1

Others expressed some resentment at the burden of having to act as administrators for their medical information. They noted that even though they owned the information, they did not want to have to assume responsibility for maintaining their medical record and providing it to different providers:

I find it very offensive...that you pay these doctors and you pay for the health care system and they have all your records...And it was like they took ownership of your life but they didn’t take responsibility.

P15

Most participants viewed the lack of a consolidated health record as the most significant challenge they faced when trying to obtain their medical records. Four expressed frustration with the burden of obtaining access to records kept by different providers.

...Specialis­­­­ts, they have their own charts for us. So even if I go to medical records, I’m not able to see or get the copy of my results because they don’t have them on the computer. So, the point is, when you go to the particular clinic and you ask for the results of the procedure or the specific test that was done in this clinic there is a problem to get the copy. I have to go to the medical records office. I fill out the form each time I want to see results. And make the trip. Then pick it up, or they send (it) to you. Some places when you ask to make a CD of your scan or something, they ask you to pay them, so you go to a different place...and it’s a lot of work for people with medical issues to do.

P3

You have to remember that the system isn’t a static thing. What they’ll give you today (PHI) may not be what they’re going to give you tomorrow, and vice versa...The other thing that’s hard to figure out is who has the power to give us what we want. It may not be the doctor. That’s not always clear. So that if you have a day where you’re seeing five different people, who’s the one who has the power to get you what you need?

P4

#### Peer Communication and Support

Participants acknowledged that new technologies offered opportunities for increased communication and support when seeking health information. Many found that people who had lived with a similar health condition offered helpful information and emotional support (eg, online support groups, patient forums, and patient ratings of hospitals or clinics) and therefore viewed them as valuable resources. For example, one person noted that online support groups offer a platform for people to share tips, not only on how to manage their condition in daily life, but also on how to navigate the health care system more easily.

You can say, “Oh, don’t go to (there) because they’ll give you the runaround. Go to this hospital.” Or “No, don’t take your child there because they do this. Go here instead.” Word of mouth and trust and people who share illnesses or have loved ones who share illnesses, are very dependent on (peer) information...It’s protecting yourself from the system, from the very system that’s there to protect you. So I think those support groups are very good for that.

P15

However, some also raised concerns about the reliability and trustworthiness of information that has been provided by other patients.

I’m not a doctor nor a physician or whatever...I read if there are suggestions, but I won’t give my knowledge because who am I? When you read something on the Internet, be careful because everybody acts like a “specialist,” so I’m hesitant on that.

P2

I went on a couple of forums...to me it showed something very clearly, it’s that so many of these people on the forums are doing this in isolation...What I (also) found was that it (the online forum) could be very easily loaded. In other words, that they would have people saying, “Oh, this is really great and wonderful software,” and then if you dug (around) it would be people that are working for the companies that were supposed to be making the software.

P4

#### Opportunities to Enhance Health Care Interactions

Participants also discussed a number of ways in which the Internet and eHealth technologies could impact the health care experience. Many viewed technology as offering an opportunity to simplify management of their health and make certain tasks more convenient (eg, prescription renewals, requesting appointments).

There’s the continuity of care, which would be like a schedule function, saying, okay, you have to see the specialist at this time, or renew your prescription, or have your prescription changed. Or even if you get a certain amount of refills around one prescription, tells you you’re down, you know, flashes, so that the next time you have to go back to a physician and get a renewal for it.

P4

Others noted that technology could potentially reduce feelings of vulnerability by allowing for more continuous monitoring of their health status and providing a way to interact directly with providers when immediate communication was needed. One participant imagined possible future scenarios:

We could take a scenario where a nurse or a doctor is watching the rates and says: “You have increased here” or “Here’s a pattern these last few days,” and they know that that indicates something might be coming on: a stroke from diabetes, or something...That’s life saving. They can email the person: “Come into the office” or “Go to the emergency room.”

P15

One participant described a recent situation where she felt upset at having to take responsibility to educate herself about her newly diagnosed medical condition.

I'm old lady and now I discovered that I have something genetic. And I ask him (the doctor), “Could you please just write the name of this so I could figure it out myself on the Internet?” (The doctor said) “I have no time. I have other patients. Did you see the corridor?” So I stood up and I turned towards the door and I said: “No. I need the name of my disease if I have to go on Internet and learn what you’re supposed to tell me yourself.”

P3

#### Privacy Concerns

The most common concerns raised about the Internet and eHealth technologies centered around privacy. All participants indicated that security of information was paramount and that they would need assurances their PHI would remain confidential before considering using any Web-based technology. For some, concerns about confidentiality appeared to outweigh potential benefits.

I wouldn’t (use the internet or eHealth technologies) because...I have a wife who works for a hospital, and they are hacked so many times I wouldn’t trust it.

P4

Never forget when you’re on the Internet, you’re not alone...So take care what you ask, take care what you do...because some people they are very, very smart on that.

P8

The issue always goes back to security, who is going to get access to your records and can records be manipulated by hackers and all that. You know...It’s one thing that hackers come into your emails, it’s horrible. But when hackers come into your financial and your medical, this is life-threatening.

P15

## Discussion

### Principal Findings

Older adults often have complex health conditions, essential self-management tasks, and frequent encounters with providers that can be facilitated with eHealth technologies. The aim of this study was to go beyond the known factors influencing eHealth uptake among older adults which include age, sex, socioeconomic status, current Internet use, and privacy concern [15,16,18-24], by exploring participants’ perceptions of these technologies in relation to their experiences with the current health care system. We found that individuals perceived there was potential value including convenience and reduced burden by using technologies that could improve access to PHI and facilitate communication between providers and peers living with similar conditions. However, we also found that acceptance of these technologies will require assurances that their PHI is in fact secure, readily accessible, and easily understood.

One of the greatest challenges consistently voiced by participants was being able to identify and access credible information about health conditions on the Web; a finding also reported by others [30-33]. Low health literacy often renders content incomprehensible [34,35]. Participants indicated that they need help in identifying information that is (1) credible, (2) unbiased, (3) easily understood, and (4) meaningful or relevant to them. There is an opportunity to develop Web-based resources to help older adults identify credible sources of information that are written in ways that make the information easy to understand. Strategies to address this include having both providers and patients review all materials prior to publication. While new methods of validating Web-based information [36] will also help to increase the credibility of information individuals receive on Web-based sites [37], additional effort is required to ensure credible tools are readily available and easily understood by older adults.

Participants had significant security and privacy concerns related to having medical information on the Web; others have noted similar concerns [18,22,24,38,39]. Although security concerns represent an important barrier to Internet and eHealth technology uptake, there is evidence that these attitudes can be changed with careful message framing. Angst and Agarwal [40] showed that privacy concerns alone are likely not sufficient to halt the acceptance of such technologies. Work continues to identify mechanisms that can help reduce the risk of unauthorized access to personal health data [41,42]. Therefore a parallel challenge is to adequately frame messages and provide the training necessary to ease users’ concerns.

Another important theme was the potential role that the Internet and eHealth technologies could play in facilitating the coordination of care services. Participants discussed the challenges of accessing medical information within their health record, which is especially important when many believed that it was ultimately going to be up to them to gather their health information and provide it to their doctors. Others have also reported on the desire of individuals to be able to access their health information [43,44].

Our results mirror one of the main findings in Ancker et al [45], in that those who have had poor experiences with accessing PHI are more likely to take on the primary responsibility for managing their own information and sharing it with their different providers.

Interestingly, a recent systematic review [46] found minimal evidence to support the notion that access to medical records resulted in improved health outcomes, however, being able to review their health-related information did enhance patients’ perception of control. Participants view the Internet and eHealth technologies as a source of convenience and a way to improve the logistics around this coordination with features allowing patients to perform transactional tasks such as booking appointments and renewing prescriptions. These administrative-type features are also highly valued by patients in the literature [33,47-49] and therefore the inclusion of these features should be considered as a mechanism for motivating individuals to use technology for long-term self-management.

The concept of who owns medical information was important to participants. Most believed they were the true owners of their health information; as such, they have the right to have ready access to it and to make it available to their other providers as needed. Empowerment is a key mechanism in the self-management of chronic diseases, particularly for older individuals [50]; therefore, leveraging this idea of ownership of health information by providing patients with access to this information could reinforce feelings of empowerment [51]. Participants in this study view eHealth as a means to gain access to their PHI but this is likely not to be sufficient to guarantee a technology’s uptake. PHRs for example have been shown to offer better access to PHI, however, evidence shows that there remain barriers to their uptake, notably that many PHRs do not include patient-oriented functionalities [52]. If technology is to be leveraged to provide easier access to PHI and, in doing so, strengthen the patients’ idea of ownership and empowerment, a patient-oriented approach to development is required to make sure that those needs are met.

Participants also discussed the value of online communities to facilitate peer support; however, several participants raised concerns about the quality and credibility of information that may be shared on social media platforms. Participants thought that the inclusion of health professionals as monitors or contributors might help offer some degree of quality control, although this approach can increase costs substantially. The question of health professionals interacting with patients on Web-based social networks requires further study in relation to privacy and legal issues [53]. One recent study looked at the use of online health communities (OHC) aimed at facilitating multidisciplinary communication among the frail and the elderly. OHCs are Internet-based applications that provide a platform uniting patients and professionals to not only share information between one another, but also to improve the coordination of care for people who have multiple caregivers. The investigators attribute an inability of the OHC to improve activities of daily living, mental health, and social activity to very low usage of the system [54].

Older adults in our study expressed interest in online communities and tools to facilitate sharing of health information and self-management strategies and the coordination of care. We also found their interest and use of the Internet and eHealth technologies to manage their health and interactions with providers are influenced by their experiences with the health care system. In particular, our study highlights the importance that patients place on the sense of ownership of their medical information, the value they place on transaction-type task (eg, booking appointments, renewing prescriptions), and how these technologies impact the health care experience. Key functionalities that participants value in eHealth products include those that (1) provide health-related information that is credible, unbiased, easily understood, and meaningful; (2) ensure security of personal medical information; (3) provide easy access to personal medical information; (4) facilitate self-coordination of care; and (5) provide access to online communities for peer support.

Despite the numerous survey-based studies aimed at elucidating factors that influence eHealth uptake among older individuals, few have evaluated how experiences with providers and the health care system work together with sociodemographic and other predictors to influence attitudes and behavior. Understanding individual differences, including how positive and negative health-related experiences impact attitudes, needs, preferences, and concerns, is essential for the development and implementation of tools in ways that encourage uptake and long-term use. However, our study has limitations. We explored the views of a convenience sample of a limited number of older adults. Participants were recruited from a large urban medical center in a system that provides universal access to health care. Future studies should explore more novel themes such as the sense of ownership of medical information, value placed on transactional tasks, and experiences with navigating the health care system. These should be explored with sample sizes large enough to understand how they fit within explanations of the digital divide experienced by older individuals. In other words, are these views merely the symptom of a cohort effect, in which case, can we expect them to change over time? Or, are they more concretely linked to aging and chronic conditions, and therefore we can expect these views to persist over time? Developing technologies with end user needs and preferences in mind is essential to ensuring that technology contributes to rather than hinders positive interactions among providers and patients they care for, and results in improved health outcomes. In the context of chronic disease management, the Internet and eHealth technologies hold potential for supporting healthy aging and patient self-management.

### Conclusions

The Internet and eHealth technologies can help older adults manage their health by giving them access to health information and a means to become a more active player in their own health care. Focus groups conducted with individuals aged 50+ years extend earlier findings regarding the influence of sociodemographic factors including age, sex, and socioeconomic status that influence interest in and use of the Internet and eHealth technology uptake. We also identified several primary needs and preferences which centered on access to PHI, security, usability, and convenience. Our results can help inform the design and implementation of Internet resources and eHealth technologies, especially for older individuals who may be less comfortable with technology use but who represent the fastest growing adopters of the Internet.

## Abbreviations

EHR: electronic health record.
OHC: online health communities.
PHI: personal health information.
PHR: personal health record.
